# Big data and computational advancements for next generation of *Microbial Biotechnology*

**DOI:** 10.1111/1751-7915.13936

**Published:** 2021-10-29

**Authors:** Zulema Udaondo

**Affiliations:** ^1^ Department of Biomedical Informatics University of Arkansas for Medical Sciences Little Rock AR USA

The exponential growth of biological data is one of the most significant trends observed in life sciences over the last few decades. Rapid technological changes and price reduction of new technologies have made omic approaches much more affordable to undertake experiments that generate vast quantities of data. In the same way, the scientific community has adopted a culture of openness which has encouraged researchers to create open‐source tools, protocols and deposit data to build massive publicly available databases designed to gather and share biological data.

We are now in a new era of biological sciences, the era of *Big (biological) data*, and along with the astonishing growth of biological data, modern computational methods and algorithms essential for the progress of experimental science have been developed to analyse high‐throughput experimental data in novel ways. However, some of these ‘novel’ methodologies, used nowadays to obtain biological knowledge leveraging large amounts of data, have been recycled from the past.

Artificial intelligence (AI), a branch of computer science devoted to building machines that are able to perform tasks that typically require human intelligence, has been around since the 1950s, but, it has recently regained popularity when the biggest companies in the world such as Google, Amazon and Uber have renovated these algorithms to power their recommendation/search engines and services.

Within the area of AI, the machine learning branch leverages large data sets and applies accurate mathematical models to teach *itself* to make more accurate predictions and classifications instead of relying on explicit programming. Thus, the main feature of machine learning algorithms is that they are able to automatically and quickly train themselves using provided data, and then they can apply this knowledge to discover hidden non‐obvious patterns in large data sets.

To further complicate the concepts a little more, we have a younger sub‐category of machine learning, deep learning, which uses multilayered neural networks (inspired by biological neural networks, called artificial neural networks) to *learn* from even larger amounts of data. This multi‐layered structure is what enables deep learning algorithms to compute very complex and abstract tasks using extremely large and complicated sets of parameters to make inferences with minimal human intervention. Using deep learning algorithms, we are now able to solve tasks that simpler machine learning models were not able to solve in the past. In the same way, deep learning is powered by Big data, and their models tend to improve their performance as the amount of training data increases. However traditional machine learning models such as Support Vector Machine and Naïve Bayes classifiers reach a saturation level that limits further improvements.

Although machine learning algorithms have been used for several decades now for prediction and discovery in different biological fields, these methodologies are still young enough to keep expanding their practical applications. For instance, machine learning algorithms have been applied since the 1980s for protein structure prediction (Qian and Sejnowski, 1988), and later to recognize specific sequences of DNA and RNA that bind proteins (Ohler *et al*., 2002) and other DNA regions of interest (Bucher, 1990; Heintzman *et al*., 2007), as well as for gene discovery (Ma *et al*., 2014). In the area of transcriptomics, machine learning algorithms were utilized for clustering gene expression patterns using microarray gene expression data (Pirooznia *et al*., 2008) and later to analyse RNA‐seq data (Thompson *et al*., 2016).

In the past few years, renovated algorithms that are replacing statistical modelling components by using deep learning models to improve their performance have become the state of the art to perform variant calling (Poplin *et al*., 2018): for accurately classifying disease‐causing variants, providing insights into the role of aberrant splicing in disease (Xiong *et al*., 2015); to predict bacteria–host associations (Martínez‐García *et al*., 2016); to recognize functional genomic elements such as enhancers and promoters (Li *et al*., 2015; Liu *et al*., 2016); and to predict the deleterious effects of nucleotide polymorphisms (Quang *et al*., 2015).

More recently, new approaches using big biological data and machine learning techniques, have been applied to discover novel chemical scaffolds for drug candidates. The traditional 'scale‐up' drug discovery process is a labour‐intensive and expensive path in which scientists extensively test millions of molecules, but only a handful of potential compounds progress to preclinical or clinical testing. However, using machine learning approaches, now we can quickly and systematically screen large compound libraries for new drug candidates. This kind of approach has already been successfully used to identify more than 300 candidate antivirals for SARS‐CoV‐2 (Huang *et al*., 2021) and was validated using already identified compounds with activity against other viruses such as Ebola and Zika. Another novelty of this approach, called biological activity‐based modelling (BABM), is that it builds on the hypothesis that compounds that show similar activity patterns tend to share similar targets or mechanisms of action and therefore the BABM approach does not require any chemical structure information of the screened compounds to make predictions. In this way BABM can be applied to any substance with available biological profiling, including macromolecules and natural products. Virtual screening campaigns of natural products and microbial secondary metabolites libraries using this and other machine learning approaches (Hannigan *et al*., 2019) could be a real game changer in the fight against multidrug‐resistant microorganisms and also in the search of novel anticancer agents.

Another striking example of machine learning applications that are helping to solve long‐term scientific challenges is the AlphaFold2 deep learning algorithm developed by Google's DeepMind Technologies. AlphaFold2 has reached a great level of popularity for obtaining the highest level of accuracy achieved to date in the latest CASP14 assessment that took place in November 2020. In the same way, the developers of AlphaFold2 claim that this algorithm is able to predict protein structures to near experimental accuracy in almost all cases (Jumper *et al*., 2021). The possibility of being able to unscramble the complete structure of proteins from their amino acid sequence in a computational and systematic way in the near future could be completely transformational for the area of *Microbial Biotechnology* in a way difficult to predict. Right now, most of the protein functions available in public repositories are obtained using sequence homology analysis which is just a *mere* approximation, as the function of the protein is actually directly determined by its tertiary structure. The ability to predict accurate protein structures from their amino acid sequence would be incredibly valuable for drug discovery, but it could even have a greater impact in other areas of biochemical engineering, for example, to enhance biocatalysts identifying key protein residues and to better estimate their optimal kinetic parameters.

A very exciting feature of this second coming of AI applied to Big biological data is that it also offers new approaches for modelling biological processes by integrating different data types (integrative multi‐omics). Machine learning approaches to mining multi‐omics information hold great promise in unravelling convoluted relationships where different biological layers interact with each other in a nonlinear manner. In multi‐omics data integration, every type of ‐omics data corresponds to one feature space (for instance, gene expression data and DNA methylation data) and it is linked through different layers of molecular feature spaces to elucidate molecular pathways underlying different conditions. Applying integrative multi‐omics, subtle changes in gene expression could be augmented using additional information from methylation analysis, for example. These novel AI approaches and methodologies could open new opportunities for obtaining better prediction performance and also to improve our understanding of the complex molecular pathways and processes that take place in microbial communities (Cai *et al*., 2021) and in bioremediation studies (Gupta *et al*., 2020). For example, an in‐depth study of the adaptation of a microbial community to a soil environment for bioremediation purposes would be unattainable when analysing single‐omics data. An integrated study of the information collected through metagenomics, transcriptomics, proteomics and metabolomics analysis would be more efficient to understand the fluctuations of the biological activities produced from the complex mechanisms of adaptation of the organisms to the new environment.

Although incredibly promising, there are still some obstacles to solve when applying deep neural networks to tackle biological problems. One of them is that as the neural network grows in layers and complexity, then it is virtually impossible to trace how all the parameters (sometimes millions of parameters) combine to make decisions. This is called the ‘black box problem’ and it means that the internal logic followed by the algorithm to generate the results has an unexplainable logic. The possibility of understanding the black box behaviour of artificial neural networks in the coming future would be a revolution in the field of AI. The way has been paved and a significant amount of work has already been done recently in the area, trying to explain how deep learning algorithms make decisions without "opening" their black box.

The anticipated potential of AI has also awoken concerns and wariness from different perspectives. Some of these concerns are related to the idea that as we get more value from computers and AI, scientists will become less important. However, we actually cannot build these complex models without human experts who can first assess which type of data is needed to be collected; second generate the appropriate data and finally, to evaluate how the model is performing.

Another important issue is that implementing successful complex deep neural network algorithms is a non‐trivial task and it has been essentially restricted so far to computational scientists and those with knowledge in the areas of programming. Fortunately, several easy‐to‐use software frameworks have been released and are now available to a broad community of researchers. Ultimately, with the recent focus of the scientific community towards methodological openness which will further bridge the gap between the general research community and machine learning experts, machine learning will become an essential toolkit of the scientists of the near future.

## Funding information

No funding information provided.

## Conflict of interest

None declared.
